# Socioeconomic impact of depression and pain in patients with neuromyelitis optica spectrum disorders

**DOI:** 10.1186/s42466-026-00486-4

**Published:** 2026-05-04

**Authors:** Daria Tkachenko, Ilya Ayzenberg, Louisa M. Schöppe, Rasmus Schülke, Friedemann Paul, Ankelien Duchow, Judith Bellmann-Strobl, Nadja Siebert, Tania Kümpfel, Joachim Havla, Hannah Pellkofer, Sven Jarius, Brigitte Wildemann, Achim Berthele, Luisa Klotz, Marc Pawlitzki, Stefan Gingele, Olivia Schreiber-Katz, Martin S. Weber, Makbule Senel, Jan-Patrick Stellmann, Vivien Häußler, Orhan Aktas, Marius Ringelstein, Kerstin Hellwig, Carolin Schwake, Ingo Kleiter, Corinna Trebst, Martin W. Hümmert

**Affiliations:** 1 Department of Neurology, Hannover Medical School, Carl-Neuberg-Straße 1, 30625 Hannover, Germany; 2 PRACTIS Clinician Scientist Program, Dean’s Office for Academic Career Development, Hannover Medical School, Hannover, Germany; 3 Ruhr University Bochum, Department of Neurology, St. Josef Hospital, Bochum, Germany; 4 Department of Psychiatry, Socialpsychiatry and Psychotherapy, Hannover Medical School, Hannover, Germany; 5 NeuroCure Clinical Research Center, Charité Universitätsmedizin Berlin, corporate member of Freie Universität Berlin, Humboldt-Universität zu Berlin, and Berlin Institute of Health, and Max Delbrück Center for Molecular Medicine, Berlin, Germany; 6 Experimental and Clinical Research Center, Charité Universitätsmedizin Berlin, corporate member of Freie Universität Berlin, Humboldt-Universität Zu Berlin, Berlin, Germany; 7 Department of Neurology, Charité Universitätsmedizin Berlin, corporate member of Freie Universität Berlin, Humboldt-Universität Zu Berlin, and Berlin Institute of Health, Berlin, Germany; 8 Institute of Clinical Neuroimmunology, LMU Hospital, Ludwig-Maximilians Universität München, Munich, Germany; 9 Department of Neurology, University Medical Center Göttingen, Göttingen, Germany; 10 Institute of Neuropathology, University Medical Center Göttingen, Göttingen, Germany; 11 Department of Translational Neuroinflammation and Automated Microscopy, Fraunhofer Institute for Translational Medicine and Pharmacology, Göttingen, Germany; 12 Molecular Neuroimmunology Group, Department of Neurology, University of Heidelberg, Heidelberg, Germany; 13 Department of Neurology, TUM School of Medicine and Health, Munich, Germany; 14 Department of Neurology, University of Münster, Münster, Germany; 15 Department of Neurology, Otto-Von-Guericke University of Magdeburg, Magdeburg, Germany; 16 Department of Neurology, University of Ulm, Ulm, Germany; 17 Department of Neurology and Institute of Neuroimmunology and MS (INIMS), University Medical Center Hamburg-Eppendorf, Hamburg, Germany; 18 Aix-Marseille Univ, CNRS, CRMBM, UMR 7339, Marseille Cedex, France; 19 APHM, Hopital de la Timone, CEMEREM, Marseille, France; 20 Department of Neurology, Medical Faculty and University Hospital, Heinrich Heine University Düsseldorf, Düsseldorf, Germany; 21 Department of Neurology, Center for Neurology and Neuropsychiatry, LVR-Klinikum, Heinrich Heine University Düsseldorf, Düsseldorf, Germany; 22 Marianne-Strauß-Klinik, Behandlungszentrum Kempfenhausen für Multiple Sklerose Kranke, Berg, Germany; 23 Department of Neurology, Klinikum Agnes Karll Laatzen / Klinikum Region Hannover, Laatzen, Germany

**Keywords:** Neuromyelitis optica spectrum disorders (NMOSD), Socioeconomic burden, Depression, Pain, Cost of illness (COI), Expanded Disability Status Scale (EDSS), Informal care costs, Formal care costs

## Abstract

**Background:**

Neuromyelitis optica spectrum disorders (NMOSD) are associated with a high burden of depression, pain, and physical disability, all of which significantly impair quality of life. At the same time, discussions on the cost-effectiveness of treatment strategies are gaining importance. However, it is not yet known whether specific symptom burdens are particularly cost-driving. This study aims to provide a comprehensive cost analysis considering depression and pain to optimise future healthcare strategies.

**Methods:**

This prospective cross-sectional multicentre study was conducted at twelve centres of the Neuromyelitis Optica Study Group (NEMOS). Over a three-year period, 115 NMOSD patients were recruited. Disease-related costs, pain, and depression were assessed using standardised questionnaires. A generalised linear model analysis and graphical sub-cost analysis were performed to identify key cost drivers. The robustness of our findings was confirmed using two independent depression rating scales.

**Results:**

In our sample of 115 patients, 77% suffered from chronic pain with a median pain intensity of 4.0 on the numeric rating scale (NRS). Moreover, 56% of patients reported depressive symptoms. In multivariate regression analysis, depression emerged as a significant predictor of total costs (p < 0.001) alongside the EDSS score (p < 0.001) and age (p = 0.004). In contrast, pain was not significantly influencing total costs (p = 0.057), despite being reported by the majority of patients. Graphical analyses highlighted informal costs as the main cost driver in patients with increasing depressive symptoms.

**Conclusions:**

Depressive symptoms are not only common in NMOSD patients but also represent a major cost driver alongside neurological disability. Addressing these symptoms is essential for optimal patient care and may help reduce the socioeconomic burden.

**Supplementary Information:**

The online version contains supplementary material available at 10.1186/s42466-026-00486-4.

## Introduction

Neuromyelitis optica spectrum disorders (NMOSD) are rare chronic inflammatory diseases of the central nervous system that characteristically manifest with spinal cord and optic nerve inflammation [1]. Relapses are characterised by severe disability, impaired spontaneous remission and often critical localisation of lesions (longitudinally extensive transverse myelitis, brainstem, chiasma opticum) [2, 3]. Invisible symptoms such as depression and pain have been shown in previous studies to be associated with the disease [4, 5]. Depression in NMOSD patients shows an overall prevalence of 46%, with about 28% scoring in the moderate to severe range [5–7]. The severity of depression is particularly linked to pain and fatigue, whereas disability status does not appear to be associated [6]. Immunologic factors such as Interleukin 17 and anti-ribosomal P-protein autoantibodies might contribute to high occurrence of depression [8–10]. Only 40% of NMOSD patients with moderate to severe depression receive treatment for this condition [6, 11]. The consequences may be enormous, including deterioration in health status, reduction in quality of life, poor psychological outcomes, and substantial economic impact [12–15]. Referring to studies of the World Health Organization, depression has the highest public health relevance of all other diseases [16].

Chronic pain occurs in 72% to 86% of NMOSD patients and the majority of them suffers from a neuropathic component [4, 11, 17]. Compared to multiple sclerosis (MS), it is more frequent and associated with lower quality of life [18, 19]. Partly it is perceived as the worst symptom, even compared to severe weakness and bladder dysfunction [18]. The economic impact of chronic pain and depression is known to be exceedingly high, even in comparison to heart diseases, hypertension, diabetes mellitus, and respiratory diseases [20, 21]. A number of studies have outlined chronic pain and depression interacting through sharing biological pathways and transmitters exacerbating one another [22].

It is likely that depression and pain affect NMOSD patients also socioeconomically. Work performance may decline, patients miss work, and career opportunities are limited. New expenses such as aids or care costs emerge [23].

Due to meanwhile approved expensive immunotherapies, discussions about economic aspects arise [24]. However, a systematic analysis of costs associated with pain and depression in NMOSD has not yet been conducted.

The aim of this study was to evaluate in detail the role of depression and pain in NMOSD patients on all types of costs from a societal perspective. We hypothesised that depression- and pain-related aspects are relevant cost-driving factors.

## Materials and methods

### Study design and study population

Patients diagnosed with NMOSD were recruited as part of two multicentre cross sectional, questionnaire-based studies carried out within the Neuromyelitis Optica Study Group (NEMOS, https://nemos-net.de/). On the one hand, the CHANCE^NMO^ Study, initiated by the Department of Neurology at Hannover Medical School, collected comprehensive and detailed socioeconomic (CHANCE^NMO^ Study Questionnaire) and quality of life (EuroQoL Group 5 Dimension 5 Level Scale questionnaire, EQ-5D-5L) data in NMOSD patients [24]. On the other hand, detailed data on pain (PainDetect, short form of Brief Pain Inventory), quality of life (Short Form 36 Health Survey, SF36), and depressive symptoms (Beck’s Depression Inventory-II, BDI-II) in NMOSD patients were collected under the coordination of the Department of Neurology of the Ruhr-University Bochum (PAIN & DEPRESSION study) [11]. The recruitment period took place from 2017 to 2019 at twelve German NEMOS centres, with a maximum interval of one year allowed between the two assessments within an individual patient, ensuring that study timeframes were nearly identical. Eligibility criteria included age ≥ 18 years and a confirmed NMOSD in accordance with the International Panel for NMO Diagnosis (IPND) 2015 criteria [2]. Exclusion criteria consisted of other serious diseases unrelated to NMOSD and severe cognitive impairment precluding informed consent. In addition, for this analysis, patients with myelin oligodendrocyte glycoprotein antibody-associated disease (MOGAD) were strictly excluded due to different underlying pathologies [25]. A total of 115 participants were included in the study after excluding those who experienced a relapse between the survey dates of the two studies.

### Standard protocol approvals and patient consents

The studies were approved by the ethics committees of Hanover Medical School (no. 2016-7217) and the Medical University of Bochum (no. 15-5534) and all patients provided their written informed consent before enrolment.

### Socioeconomic aspects

Socioeconomic data were captured using the data from the CHANCE^NMO^ Study. For details on the methodology of cost estimation, please refer to Hümmert et al*.* [24]. In brief, the detailed questionnaire covers all types of annual costs from a societal perspective, with the following cost categories: direct medical costs, direct non-medical costs, and indirect costs, which account for the total annual cost of illness (COI) in Euros for 2018 (main recruitment period; 2018 average: €1 = US dollar [USD] 1.18). For the calculation of direct medical costs, information on treatment (general practitioner, outpatient treatment, hospitalisation and rehabilitation due to NMOSD), formal care, type of medication and the associated out-of-pocket co-payment in each case was requested and calculated according to insurance status. Furthermore, remedies and medical aids (mobility aids, everyday help, care aids), which were also assigned to direct medical costs, were surveyed and calculated. Direct non-medical costs are composed of investments due to necessary environmental changes (e. g. house and car modifications) because of the illness, informal care costs (care provided by untrained personnel such as relatives or friends), and transportation costs. Indirect costs refer to the loss of productivity of patients (salary loss due to reduction of working time, sick leaves, unemployment, and early retirement).

### Assessment of pain

The questions of the short form of the Brief Pain Inventory were based on a numeric rating scale (NRS) from 0 (no pain) to 10 (worst pain imaginable) within the last week and consisted of two categories—pain severity and interference with daily life. Interference was rated from 0 (no interference) to 10 (complete interference). General activity, mood, walking ability, working ability, relations with other people, sleep, and enjoyment of life were considered. The PainDetect Questionnaire was administered to ask about pain localization and to distinguish between neuropathic and nociceptive pain. Scores range from −1 to 38, with scores from 19 to 38 classified as neuropathic, scores from 13 to 18 as possibly neuropathic, and scores from −1 to 12 as nociceptive. Prior to completing the questionnaires, patients were instructed to report exclusively pain that they attributed to NMOSD.

### Assessment of depression

Depressive symptoms were assessed using two different types of questionnaires. First, assessment was performed with the BDI-II. The score ranges from 0 (best) to 63 (worst): < 9: no depressive affect; 9–13: minimal mood disturbance; 14–20: mild depressive symptoms, 21–28: moderate depressive symptoms; ≥ 29: severe depressive symptoms. A score of 14 and above is considered clinically relevant depressive symptoms. To cross-check our results, we used the fifth dimension (anxiety/depression) of the EQ-5D-5L questionnaire, a validated questionnaire to assess health-related quality of life. This dimension measures depression/anxiety on a score ranging from 1 (I am not depressed or anxious), 2 (slight problems), 3 (moderate problems), 4 (severe problems) and 5 (unable to perform daily usual activities due to anxiety and/or depression). Questionnaires are used as screening tools to identify individuals who may have increased symptom burden; they are not a substitute for a diagnostic evaluation by a clinician and cannot be used to establish a definitive diagnosis [26].

### Assessment of quality of life

Quality of life was measured using two questionnaires: First, the Short Form 36 Health Survey (SF-36), which consists of 36 items that can be divided in 8 subscales with components of mental and physical health. Each of the 8 subscales is then transformed into a range from 0 to 100. Lower scores indicate higher degree of disability and vice versa. Second, the EuroQoL Group 5 Dimensions 5 Level Scale (EQ-5D-5L) questionnaire, which asks for the level of problems (rated from 0 no problems to 5 unable/extreme problems) in 5 dimensions including mobility, self-care, management of usual activities, pain/discomfort, and anxiety/depression.

### Statistical analysis

The statistical analysis was carried out with IBM Statistics 29.0 and generalised linear model analysis was carried out with R version 4.2.2. Comparisons of socio-demographic and medical characteristics of the participants were presented in absolute frequencies, percentages, median and range. Relevant variables of both studies (CHANCE^NMO^ and PAIN & DEPRESSION study aspects) were included in the analysis. Given the skewed nature of cost variable(s), we performed generalised linear models (GLM), anticipating that the dependent variable(s) follow a gamma rather than a normal distribution, to identify whether depressive symptoms (measured by BDI-II and EQ-5D-5L anxiety/depression dimension) and/or pain were independent influencing factors/determinants of disease costs. Depressive symptoms detected by GLM analysis as an independent cost-driving factor were visualised by boxplot analysis, in which outliers were determined. Subsequently, bar graphs were used to identify important cost categories. Depression as the important cost-driving factor was verified using the EQ-5D (measurement of anxiety/depression). Statistical significance was set at p < 0.05.

## Results

### Characteristics of the study cohort

During a recruitment period from 2017 to 2019, 115 participants were enrolled in both studies in twelve tertiary referral centres (for details on sample characteristics, see Table 1). The majority were women (98 patients, 85%) with a median age of 53 years and predominantly aquaporin 4-antibody positive (93%). The EDSS score ranged from 0–8.5, with a median of 3.5. In the overall societal cost assessment (CHANCE^NMO^ study parameters), the average total COI was calculated at €56,299 (95% CI 44,778–67,820, or USD 60,971, 95% CI 48,497–73,452) per patient per year. Informal care costs were the largest contributor to the total COI with €17,879. (95% CI 12,899–22,859, or USD 19,364, 95% CI 13,970–24,757). Almost every fourth patient (28 patients, 24%) was on early retirement. The loss of productivity amounted to an average indirect cost of €13,383 (95% CI 7,417–19,348, or USD 14,490, 95% CI 8,030–20,949) per patient per year.

**Table 1:** Sample characteristics

		Median (IQR) / (min. – max.)
*Demographic characteristics*		
Total number of patients		115
Female Sex, n (%)		98 (85)
Age, in years		53 (42–61)
*Clinical characteristics*		
EDSS		3.5 (2–5)/ 0–8.5
Disease duration, in years		7 (3–12)
Serostatus		AQP4-IgG^+^ (n = 107; 93%) Double seronegative (n = 8; 7%)
*CHANCE*^*NMO*^* study parameters, Mean (95%CI)*		
Total annual cost of illness, €		56,299 (44,778–67,820)
Annual informal care costs, €		17,879 (12,899–22,858)
Working patients, n (%)		72 (63)
Indirect costs, €		13,383 (7,414–19,348)
Early-retirement, n (%)		28 (24)
EQ-5D-5L score		2 (1,9–2,2) /1–4
*PAIN & DEPRESSION study parameters*		
Patients with pain, n (%)		88 (77)
Median pain intensity according to NRS		4 (2–5)
Pain quality	Nociceptive, n (%)	28 (25)
	Probable neuropathic, n (%)	27 (24)
	Neuropathic, n (%)	33 (30)
BDI-II score*		10 (6–17)/ 0–46
Depressive symptoms	None, n (%)	50 (44)
	Minimal, n (%)	20 (17)
	Mild, n (%)	23 (20)
	Moderate, n (%)	12 (11)
	Severe, n (%)	9 (8.0)
*SF-36*		
SF-36 physical composite		35 (27–43)/ 14–59
SF-36 mental composite		51 (40–57)/ 15–62

IQR = Interquartile Range; CI = Confidence Interval; AQP4-IgG = aquaporin-4-immunoglobulin G; BDI-II = Beck’s Depression Inventory-II; EDSS = Expanded Disability Status Scale; NRS = numeric rating scale with values from 0 to 10 (0 no pain, 10 worst pain imaginable). *BDI-II total score available for 114 of 115 patients.

Most patients (77%) reported to suffer from chronic NMOSD-related pain. 27 (23%) patients reported no NMOSD-related pain. The median pain intensity on the numeric analogue scale was 4.0 (range 2–5). The distribution of pain types was nearly even across nociceptive (25%), probable neuropathic (24%), and definite neuropathic (30%) categories. Depressive symptoms assessed by the BDI-II were distributed as follows: 50 participants (44%) reported no depressive symptoms, 43 (37%) minimal or mild depressive symptoms, and 21 (19%) moderate or severe depressive symptoms (Table 1). In the self-assessment of quality of life of the SF-36 questionnaire, the median score on the mental composite was 51 (minimal 15–maximal 62) and the reported physical composite score was reported with a median score of 35 (minimal 14–maximal 59). Both scores are substantially below the median of the general population [27].

### Drivers of total cost of illness

To identify the cost-driving factors in NMOSD patients and based on the right skewness of the cost data a generalised linear model analysis was conducted. The independent predictors of total COI were EDSS F(1,85) = 20,462 (p < 0.001), age F(1,83) = 8,689 (p = 0.004), and BDI score F(4,76) = 5,789 (p < 0.001) (Table 2). To prove these results, the same analysis was performed using a different measurement for depressive symptoms: The depression/anxiety dimension of the EQ-5D-5L questionnaire (Table 3). Applying generalised linear model analysis, it was again shown that the extent of depressive symptoms, now expressed by the fifth dimension of the EQ-5D-5L, was an independent predictor of total COI (F(3,75) = 3,915 (p < 0.05)). In contrast, neither pain intensity nor pain quality proved to be significant in these two models (Tables 2 and 3). Conclusively, depressive symptoms were found to have a significant impact on the total COI in NMOSD, regardless of the EDSS and age of patients.

**Table 2:** Predictors of total cost of illness

Total annual cost vs.	df_numerator_	df_denominator_	F	*p*
EDSS	1	85	20,462	** < 0.001**
Sex	1	84	1,342	0.250
Age	1	83	8,689	**0.004**
Duration of illness	1	82	0,082	0.776
Mean pain intensity	1	81	3,729	0.057
Quality of pain	1	80	0,029	0.866
Degree of depressive symptoms (BDI-II)	4	76	5,789	** < 0.001**

EDSS = Expanded Disability Status Scale; BDI-II = Beck’s Depression Inventory-II; df_numerator =_ degrees of freedom numerator; df_denominator_ = degrees of freedom denominator; F = F-Test. Significant values are shown in bold.

**Table 3:** Predictors of total cost of illness (depression/anxiety dimension of the EQ-5D-5L instead of the BDI-II)

Total annual cost vs.	df_numerator_	df_denominator_	F	*p*
EDSS	1	83	17,112	** < 0.001**
Sex	1	82	1,246	0.268
Age	1	81	7,738	**0.007**
Duration of illness	1	80	0,119	0.731
Mean pain intensity	1	79	2,647	0.108
Quality of pain	1	78	0,026	0.873
Level of depressive symptoms (EQ-5D-5L)	3	75	3,915	**0.012**

EQ-5D-5L = European Quality of Life 5 Dimensions Level Version; EDSS = Expanded Disability Status Scale; df_numerator =_ degress of freedom numerator; df_denominator_ = degress of freedom denumerator; F = F-Test. Significant values are shown in bold.

### Cost-driving subcomponents of depressive symptoms

**Fig. 1:**
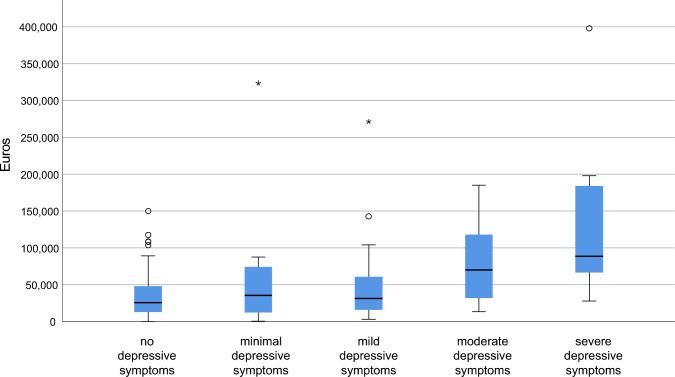
Mean annual total cost of illness per patient stratified by depressive symptoms (BDI-II). BDI-II scores were interpreted using the following cutoff values: none depressive symptoms 0–8; minimal depressive symptoms 9–13; mild depressive symptoms 14–20; moderate depressive symptoms 21–28; severe depressive symptoms 29–63.

**Fig. 2:**
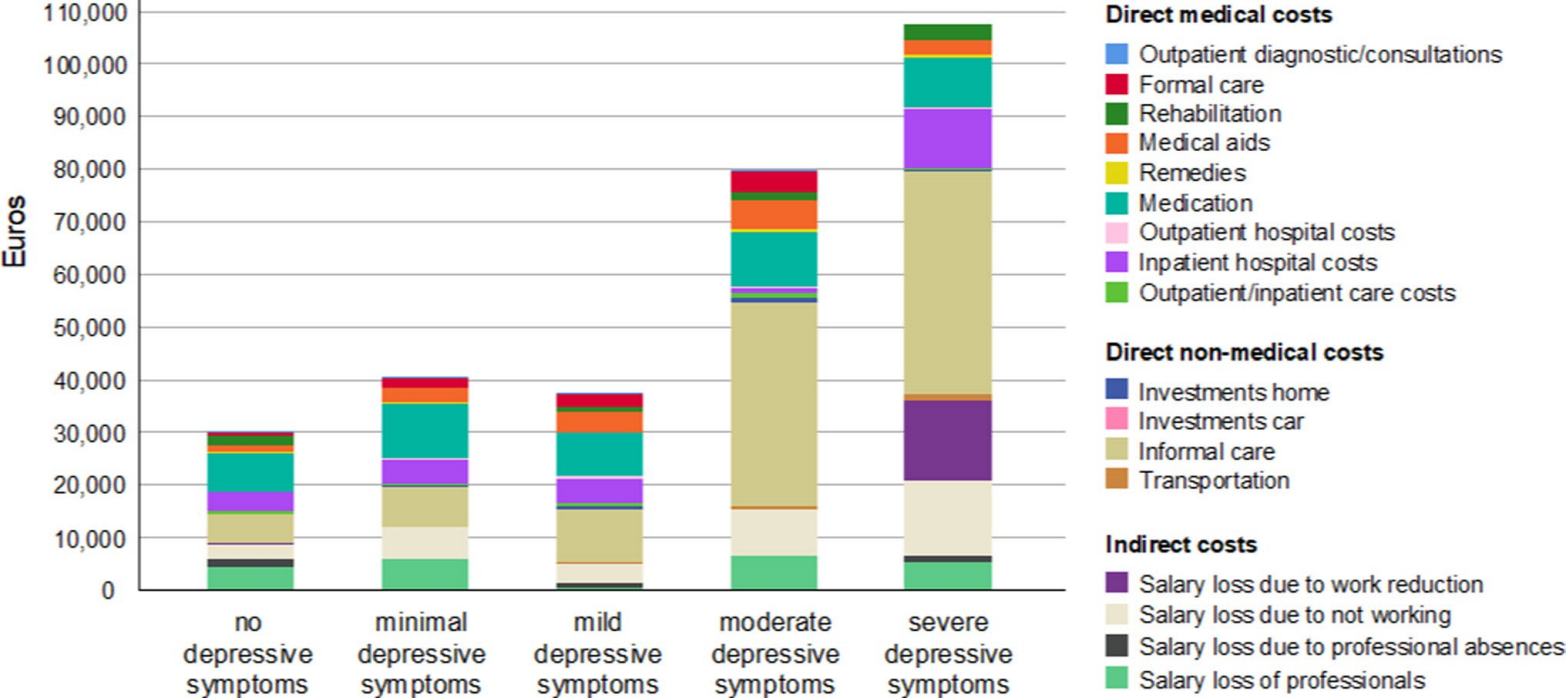
Mean annual total cost of illness per patient stratified by depressive symptoms (BDI-II). BDI-II scores were interpreted using the following cutoff values: none depressive symptoms 0–8; minimal depressive symptoms 9–13; mild depressive symptoms 14–20; moderate depressive symptoms 21–28; severe depressive symptoms 29–63.

**Fig. 3:**
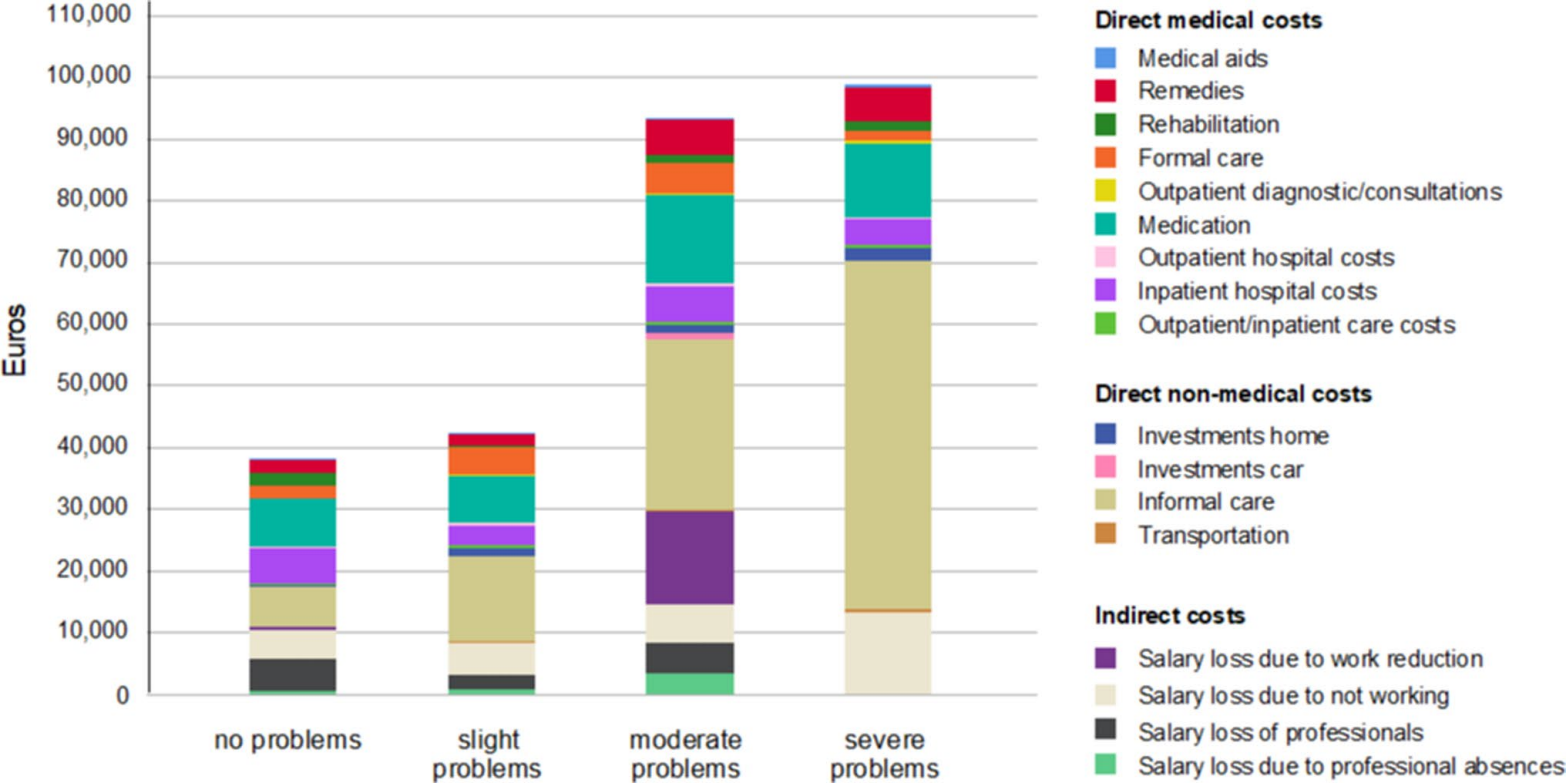
Mean annual total cost of illness per patient stratified by indication of anxiety / depression in EQ-5D-5L questionnaire. Patients were able to provide information on a scale of 0 to 5 (0 = no problems, 5 = unable/extreme problems) for each of the 5 dimensions of the EuroQoL 5 Dimensions 5 Levels questionnaire. Since no patient indicated extreme problems (level 5) in the anxiety/depression dimension, the presentation is scaled to level 1 (no problems) to level 4 (severe problems).

Boxplot analysis (Fig. 1) showed that the total COI increased as a function of the severity of depressive symptoms (BDI-II), providing plausibility for our generalised linear model analyses. A bar chart was additionally generated to illustrate the amount of the different cost categories divided in direct medical costs, direct non-medical costs, and indirect costs, depending on the level of depressive symptoms (Fig. 2 stratified by BDI-II, Fig. 3 stratified by EQ-5D-5L). After exclusion of outliers based on boxplot analyses, the main cost drivers were identified and visualized using bar graphs. Outliers were identified using the interquartile range (IQR) method, excluding observations lying more than 1.5 × IQR below the first or above the third quartile. Thus, informal care costs were found to rise with increasing degree of depressive symptoms (eTables 1–5, respectively), with informal care costs without relevant depressive symptoms of €10,133 (95% CI 4,708–15,558, or USD 11,398, 95% CI 5,297–17,503) and informal care costs with severe depressive symptoms of €42,291 (95% CI 15,191–69,391, or USD 47,584, 95% CI 16,435–75,076). No other types of COI, including formal care costs, were associated with severity of depression measured either with BDI (Fig. 2) or EQ-5D-5L (Fig. 3).

## Discussion

The key and highly relevant finding of our combined multicentre study approach is that depressive symptoms, along with severity of the disease (measured by EDSS) and age, are the main disease-related cost-driving factors in NMOSD patients. These results were proven using two different types of measurements (BDI-II and EQ-5D-5L) [28]. Depression is well-known to be the most common mental disorder in our society [29]. The Lancet commissioned Global Burden of Disease studies show that mental disorders, especially depression and anxiety, are the leading cause of disability globally and is estimated to cost the global economy 16 trillion USD by 2030 [30]. Depression as a comorbidity, given its high prevalence in NMOSD, may therefore represent an even greater economic burden. Consequently, screening NMOSD patients for depressive symptoms and ensuring adequate treatment not only has the potential to improve patients’ quality of life, but also serves as a crucial resource for reducing healthcare costs [31]. According to the Germany Barometer of Depression, it takes an average of 20 months for individuals with depression to seek professional help [32]. By that time, the condition is already advanced, more challenging to treat, and associated with higher healthcare costs [33]. Therefore, early education and therapeutic interventions should be particularly emphasised in the context of NMOSD. Although patients with NMOSD are usually under neurological care, diagnostic and therapeutic delays may still occur, largely due to a lack of awareness.

Informal care costs have been revealed as the most relevant cost driver in the context of depressive symptoms. This aligns with findings from previous studies on depression. According to the World Health Organisation it is supposed to be one of the most frequent causes leading to disability when coexisting with other medical conditions [34, 35]. People are more likely to miss time from work and a lack of motivation leads to the inability of performing domestic tasks [35–37]. A study investigating informal caregiving for older Americans with depressive symptoms revealed exceptionally high time and cost burdens [35]. Similarly, research on MS patients and their caregiving partners showed a considerable impact on caregivers, with increased strain and responsibility [38]. The cross-sectional design of our study prevents the establishment of causal relationships. Thus, it remains unclear whether depression drives the high need for informal care and costs, or whether increased caregiving demands exacerbate depressive symptoms by, for example, disrupting interpersonal relationships. Other factors such as disease severity, fatigue, loss of independence, or social isolation may also contribute to this complex interaction. Given that partners often assume the caregiver role, this dynamic is particularly relevant. Both directions of influence have been described—depressive symptoms can shape the perception of care, while caregiver behaviour impacts depression [39, 40]. Longitudinal studies are needed to disentangle these bidirectional relationships, assessing how changes in caregiving demands influence depressive symptoms over time and vice versa, while also accounting for the moderating role of social support and the specific dynamics within families of NMOSD patients.

Compared to informal care costs, formal care costs are low and show no association with depression. However, this may indirectly indicate an unmet need for professional support. Depressive patients often attempt to manage their difficulties alone or within their families for an extended period before seeking professional help, by which time their condition has often worsened [33, 41]. The predominance of informal over formal care underscores the need for improved access to information and support services, which could also yield economic benefits. Future studies should explore whether early identification and targeted management of depressive symptoms in NMOSD may lead to a reduction in reliance on informal caregiving. Demonstrating such effects would provide important insight into the potential economic benefits of structured depression management in NMOSD. Further research into caregiver burden may help identify gaps in care and inform targeted interventions.

Although chronic pain was prevalent in our cohort, affecting 77% of patients, it did not emerge as an independent cost-driving factor in the multivariable analyses. This finding may appear unexpected given the well-documented burden of pain in NMOSD and its known impact on quality of life [11]. However, pain may exert its socioeconomic effects indirectly, for example through its close association with depressive symptoms or disability. Nevertheless, given its high prevalence and clinical relevance, pain remains an important therapeutic target in NMOSD, even if its direct economic impact appears less pronounced than that of depressive symptoms in our model.

This study has several limitations. The cross-sectional design precludes causal inferences, highlighting the need for longitudinal studies to validate the findings and provide a deeper understanding of the observed associations. Economic data were based on self-reported resource utilisation, including retrospective assessments, making them susceptible to recall bias. Additionally, pain and depressive symptoms were measured through self-report, introducing a potential risk of socially desirable responses that may distort reality. We did not assess sleep disorders or fatigue, which are common in NMOSD and may represent contributors or confounders of depressive symptom severity [42]. Pain was assessed by BPI and PainDETECT, and our models may have lacked power to detect independent pain-related cost effects, including interference with indirect pathways via depression or disability, which may have contributed to pain missing statistical significance.

However, this study has notable strengths. Given the rarity of NMOSD, the sample size of 115 patients is substantial. The high recruitment rates—79% in the CHANCE^NMO^ Study and 83% in the Pain and Depression study—demonstrate strong patient engagement, highlighting the perceived relevance of the topic. Furthermore, the central findings were validated using two independent depression measures, both yielding similar associations, which reinforces the robustness and reliability of our data.

## Conclusions

In summary, this study highlights the significant economic impact of depression among NMOSD patients, particularly in relation to informal care costs. Beyond confirming the high prevalence of depression in this population, our findings emphasise its role as a major cost driver. These results highlight the urgent need for better awareness and further research on informal care and its impact on patients’ mental well-being. Routine screening for depression and treatment needs should be integrated into clinical assessments of NMOSD patients. Future studies should investigate whether increased professional support could alleviate depressive symptoms while simultaneously reducing overall costs. Targeted interventions could help optimise patient care and assess the extent to which effective management of depressive symptoms contributes to lowering healthcare expenditures. Chronic pain was common in our cohort, even if no independent association with total costs was observed.

## Supplementary Information

Additional file 1.

## Data Availability

Anonymised datasets not published within the article will be shared by the corresponding author upon reasonable request by any qualified investigator.

## Appendix

**Collaborators The Neuromyelitis Optica Study Group (NEMOS):** Philipp Albrecht, MD, Kliniken Maria Hilf, Mönchengladbach, Germany; Klemens Angstwurm, MD, University of Regensburg, Regensburg, Germany; Susanna Asseyer, MD, Charité Universitätsmedizin, Berlin, Germany; Antonios Bayas, MD, University Hospital, Augsburg, Germany; Natalie Bednarz, MD, University Hospital, Leipzig, Germany; Stefanie Behnke, MD, Knappschaftsklinikum Saar, Sulzbach, Germany; Stefan Bittner, MD, University Medical Center of the Johannes Gutenberg University, Mainz, Germany; Franziska Bütow, MD, Hannover Medical School, Hannover, Germany; Mathias Buttmann, MD, Caritas-Krankenhaus, Bad Mergentheim, Germany; Eva Dawin, University Hospital, Münster, Germany; Rick Dersch, MD, University Hospital, Freiburg, Germany; Daniel Engels, MD, LMU University Hospital, LMU Munich, Munich, Germany; Thorleif Etgen, MD, Kliniken Südostbayern, Traunstein, Germany; Carsten Finke, MD, Charité Universitätsmedizin, Berlin, Germany; Katinka Fischer, University Hospital, Düsseldorf, Germany; Moritz Förster, MD, Kliniken Maria Hilf, Mönchengladbach, Germany; Mathias Fousse, MD, University Hospital, Homburg, Germany; Benedikt Frank, MD, University Hospital, Essen, Germany; Frank Freitag, MD, Nervenzentrum, Potsdam, Germany; Anna Gahlen, MD, Ruhr University of Bochum, Germany; Achim Gass, MD, University Hospital, Mannheim, Germany; Johannes Gehrig, MD, University Hospital, Frankfurt, Germany; Christian Geis, MD, Jena University Hospital, Germany; Katrin Giglhuber, MD, Technical University Munich, Klinikum rechts der Isar, Munich, Germany; Yasemin Goereci, MD, University Hospital, Cologne, Germany; Ralf Gold, MD, Ruhr University of Bochum, Germany; Ana Beatriz Ayrosa Galvao Ribeiro Gomes, MD, University Hospital, Basel, Switzerland; Jonas Graf, MD, University Hospital, Düsseldorf, Germany; Sergiu Groppa, MD, University Medical Center of the Johannes Gutenberg University, Mainz, Germany; Julia Gutbrod, MD, University Hospital, Augsburg, Germany; Kerstin Guthke, MD, Klinikum Görlitz, Germany; Axel Haarmann, MD, University of Würzburg, Würzburg, Germany; Maria Hastermann, MD, Charité Universitätsmedizin, Berlin, Germany; Bernhard Hemmer, MD, Technical University, Munich, Germany; Mariella Herfurth, University Hospital, Leipzig, Germany; Marina Herwerth, MD, University Hospital, Zurich, Switzerland; Frank Hoffmann, MD, Krankenhaus Martha-Maria, Halle, Germany; Olaf Hoffmann, MD, St. Josefs-Krankenhaus, Potsdam, Germany; Ulrich Hofstadt-van Oy, MD, Klinikum Westfalen, Dortmund, Germany; Leila Husseini, MD, University Medical Centre Göttingen, Göttingen, Germany; Kathleen Ingenhoven, University Hospital, Düsseldorf, Germany; Jutta Junghans, MD, Krankenhaus Martha-Maria, Halle, Germany; Matthias Kaste, MD, Nordwest Hospital Sanderbusch, Sande, Germany; Karsten Kern, MD, Knappschaftsklinikum Saar, Sulzbach, Germany; Peter Kern, MD, Asklepios Klinik, Teupitz, Germany; Pawel Kermer, MD, Nordwest Hospital Sanderbusch, Sande, Germany; Christoph Kleinschnitz, MD, University Hospital, Essen, Germany; Wolfgang Köhler, MD, University Hospital, Leipzig, Germany; Kimberly Körbel, MD, University Hospital, Frankfurt, Germany; Markus Kowarik, MD, University Hospital, Tübingen, Germany; Markus Krämer, MD, Alfried-Krupp-Krankenhaus, Essen, Germany; Markus Krumbholz, MD, University Hospital Brandenburg, Rüdersdorf, Germany; Julian Reza Kretschmer, Hannover Medical School, Hannover, Germany; Volker Kunzmann, MD, Nervenzentrum, Potsdam, Germany; Natalia Kurka, MD, University Hospital, Frankfurt, Germany; Theodoros Ladopoulos, Ruhr University of Bochum, Germany; Andrea Landwehr, University Hospital, Münster, Germany; Stefan Langel, MD, Landeskrankenhaus Rheinhessen, Germany; Ann Sophie Lauenstein, MD, Helios Klinikum, Wiesbaden, Germany; Sarah Laurent, MD, University Hospital, Cologne, Germany; Frank Leypoldt, MD, Christian-Albrechts-University Kiel, Kiel, Germany; De-Hyung Lee, MD, University Hospital, Regensburg, Germany; Dominik Lehrieder, MD, University of Würzburg, Würzburg, Germany; Martin Liebetrau, MD, St. Josefs-Hospital, Wiesbaden, Germany; Gero Lindenblatt, MD, University Hospital, Düsseldorf, Germany; Ralf Linker, MD, University Hospital, Regensburg, Germany; Lisa Lohmann, MD, University Hospital, Münster, Germany; Felix Lüssi, MD, University Medical Centre of the Johannes Gutenberg University Mainz, Mainz, Germany; Peter Lüdemann, MD, Agaplesion Ev. Bathildiskrankenhaus, Bad Pyrmont, Germany; Michelle Maiworm, MD, University Hospital, Frankfurt, Germany; Martin Marziniak, MD, Isar-Amper Klinik Ost, Munich, Germany; Christoph Mayer, MD, Neurologischen Gemeinschaftspraxis im Bienenkorbhaus, Frankfurt, Germany; Stefanie Meister, MD, University Hospital, Rostock, Germany; Arthur Melms, MD, Facharztpraxis für Neurologie und Psychiatrie, Stuttgart, Germany; Mathias von Mering, MD, Klinikum Bremen-Nord, Bremen, Germany; Imke Metz, MD, University Hospital, Göttingen, Germany; Sven Meuth, MD, University Hospital, Düsseldorf, Germany; Jasmin Naumann, MD, Knappschaftsklinikum Saar, Sulzbach, Germany; Marjan Nenkov, University Hospital, Regensburg, Germany; Oliver Neuhaus, MD, SRH Krankenhaus, Sigmaringen, Germany; Tradite Neziraj, MD, University Hospital, Basel, Switzerland; Moritz Niederschweiberer, Charité Universitätsmedizin, Berlin, Germany; Sabine Niehaus, MD, Klinikum Dortmund, Germany; Frederike Cosima Oertel, MD, Charité Universitätsmedizin, Berlin, Germany; Carolin Otto, MD, Charité Universitätsmedizin, Berlin, Germany; Florence Pache, MD, Charité Universitätsmedizin, Berlin, Germany; Thivya Pakeerathan, Ruhr University of Bochum, Germany; Sandra Paryjas, University Medical Center of the Johannes Gutenberg University, Mainz, Germany; Sulyn Pepping, Charité Universitätsmedizin, Berlin, Germany; Steffen Pfeuffer, MD, University Hospital, Giessen, Germany; Mosche Pompsch, MD, Alfried-Krupp-Krankenhaus, Essen, Germany; Roxanne Alice Pretzsch, University Hospital, Basel, Switzerland; Anne-Katrin Proebstel, MD, University Hospital, Basel, Switzerland; Maria Protopapa, MD, University Medical Center of the Johannes Gutenberg University, Mainz, Germany; Hans-Ulrich Puhlmann, MD, Schlosspark-Klinik, Berlin, Germany; Refik Pul, MD, University Hospital, Essen, Germany; Sebastian Rauer, MD, University Hospital, Freiburg, Germany; Torsten Rehfeldt, MD, Dietrich Bonhoeffer Klinikum, Neubrandenburg, Germany; Nele Retzlaff, University Hospital, Rostock, Germany; Arne Riedlinger, MD, Asklepios Klinik, Teupitz, Germany; Paulus Rommer, MD, Medical University of Wien, Austria; Kevin Rostásy, MD, Vestische Caritas-Kliniken, Datteln, Germany; Veith Rothhammer, MD, University Hospital, Erlangen, Germany; Lioba Rückriem, MD, MediClin Hedon-Klinik, Lingen (Ems), Germany; Klemens Ruprecht, MD, Charité Universitätsmedizin, Berlin, Germany; Christoph Ruschil, MD, University Hospital, Tübingen, Germany; Carina Saggau, University Hospital, Kiel, Germany; Patrick Schindler, MD, Charité Universitätsmedizin, Berlin, Germany; Muriel Schraad, University Medical Center of the Johannes Gutenberg University, Mainz, Germany; Matthias Schwab, MD, Jena University Hospital, Jena, Germany; Patricia Schwarz, MD, University Hospital, Tübingen, Germany; Maria Seipelt, MD, University Hospital, Marburg, Germany; Jörn Peter Sieb, MD, Helios Hanseklinikum, Stralsund, Germany; Gilberto Soloroza, MD, Charité Universitätsmedizin, Berlin, Germany; Claudia Sommer, MD, University Hospital, Würzburg, Germany; Alexander Stefanou, MD, Katharinenhospital Stuttgart, Germany; Andrea Steinbrecher, MD, Helios Klinikum, Erfurt, Germany; Heike Stephanik, MD, University Hospital, Magdeburg, Germany; Verena Steuerwald, MD, University Hospital, Augsburg, Germany; Muriel Stoppe, MD, University Hospital, Leipzig, Germany; Klarissa Stürner, MD, University Hospital, Kiel, Germany; Marie Süße, MD, University Hospital, Greifswald, Germany; Florian Then Bergh, MD, University of Leipzig, Leipzig, Germany; Athanasios Tarampanis, University Hospital, Düsseldorf, Germany; Simone Tauber, MD, University Hospital, Aachen, Germany; Thanos Tsaktanis, MD, University Hospital, Erlangen, Germany; Hayrrettin Tumani, MD, University Hospital, Ulm, Germany; Ulrike Wallwitz, MD, Martha-Maria Hospital Halle-Doelau, Halle (Saale), Germany; Clemens Warnke, MD, Faculty of Medicine and University Hospital Cologne, University of Cologne, Cologne, Germany; Klaus-Peter Wandinger, MD, University Medical Center Schleswig–Holstein Campus, Lübeck, Germany; Jens Weise, MD, Helios Vogtland-Klinikum, Plauen, Germany; Jonathan Wickel, MD, Jena University Hospital, Germany; Heinz Wiendl, MD, University Hospital, Münster, Germany; Alexander Winkelmann, MD, University Hospital, Rostock, Germany; Felix Wohlrab, MD, Charité Universitätsmedizin, Berlin, Germany; Yavor Yalachkov, MD, University Hospital, Frankfurt, Germany; Clarissa Zappe, MD, Technical University Munich, Klinikum rechts der Isar, Munich, Germany; Uwe Zettl, MD, University Hospital, Rostock, Germany; Ulf Ziemann, MD, University Hospital, Tübingen, Germany; Frauke Zipp, MD, University Medical Center of the Johannes Gutenberg University, Mainz, Germany.

## Abbreviations

NMOSD: Neuromyelitis optica spectrum disorders
MS: Multiple sclerosis
NEMOS: Neuromyelitis Optica Study Group
EQ-5D-5L: EuroQoL Group 5 Dimension 5 Level Scale questionnaire
PainDetect: Short form of Brief Pain Inventory
SF36: Short Form 36 Health Survey
BDI: Beck’s Depression Inventory
IPND: International Panel for NMO Diagnosis
MOGAD: Myelin oligodendrocyte glycoprotein antibody-associated disease
COI: Total annual cost of illness
NRS: Numeric rating scale
EDSS: Expanded Disability Status Scale
GLM: Generalised linear models
AQP4-IgG: Aquaporin-4-immunoglobulin G
CI: Confidence Interval
IQR: Interquartile range
M: Mean
Md: Median
EUR: Euro
USD: US dollar
