# Supplementary material for: Socioeconomic impact of depression and pain in patients with neuromyelitis optica spectrum disorders
Source: Neurol Res Pract. 2026 May 4;8(1):36. doi: 10.1186/s42466-026-00486-4 (PMC13141397; doi:10.1186/s42466-026-00486-4)
Supplement: Supplementary file 1 — Additional file 1. [file 42466_2026_486_MOESM1_ESM.docx]

# SUPPLEMENT

**eTable 1** Sample characteristics- no depressive symptoms

|  | | **Median (IQR) / (min. – max.)** |
| --- | --- | --- |
| **Demographic characteristics** | |  |
| Total number of patients | | 50 |
| Female Sex, n (%) | | 39 (78) |
| Age, in years | | 54 (41-59) |
| **Clinical characteristics** | |  |
| EDSS | | 3 (2-4)/ 0 - 8 |
| Disease duration, in years | | 6 (4-13) |
| Serostatus | | AQP4-IgG^+^ (n=46; 92%)  Double seronegative (n=4; 8%) |
| **CHANCE^NMO^ study parameters, Mean (95%CI)** | |  |
| Total annual cost of illness, € | | 38,727 (28,640-48,815) |
| Annual informal costs, € | | 10,133 (4,708-15,558) |
| Working patients, n (%) | | 22 (41) |
| Indirect costs, € | | 10,111 (5,495-14,727) |
| Early-retirement, n (%) | | 13 (26) |
| **PAIN & DEPRESSION study parameters** | |  |
| Patients with pain, n (%) | | 33 (66) |
| Median pain intensity according to NRS | | 2.0 (1-4) |
| Pain quality | nociceptive, n (%)  probable neuropathic, n (%)  neuropathic, n (%) | 14 (28)  12 (24)  8 (16) |
| **SF-36** | |  |
| SF-36 physical composite | | 43 (33-53)/ 18-59 |
| SF-36 mental composite | | 55 (51-58)/ 32- 62 |

Abbreviations: IQR = Interquartile Range; CI = Confidence Interval; AQP4-IgG = aquaporin-4-immunoglobulin G; BDI-II = Beck´s Depression Inventory-II; EDSS = Expanded Disability Status Scale; NRS = numeric rating scale with values from 0 to 10 (0 no pain, 10 worst pain imaginable).

**eTable 2** Sample characteristics- minimal depressive symptoms

|  | | **Median (IQR) / (min. – max.)** |
| --- | --- | --- |
| **Demographic characteristics** | |  |
| Total number of patients | | 20 |
| Female Sex, n (%) | | 18 (90) |
| Age, in years | | 52 (42-65) |
| **Clinical characteristics** | |  |
| EDSS | | 3.5 (3-5)/ 2 - 8 |
| Disease duration, in years | | 7.5 (3-14) |
| Serostatus | | AQP4-IgG^+^ (n=20; 100%) |
| **CHANCE^NMO^ study parameters, Mean (95%CI)** | |  |
| Total annual cost of illness, € | | 56,039 (23,474-88,603) |
| Annual informal costs € | | 12,577 (2,085-23,069) |
| Working patients, n (%) | | 9 (45) |
| Indirect costs, € | | 13,113 (5,341-20,884) |
| Early-retirement, n (%) | | 7 (35) |
| **PAIN & DEPRESSION study parameters** | |  |
| Patients with pain, n (%) | | 19 (95) |
| Median pain intensity according to NRS | | 3.0 (3-5) |
| Pain quality | nociceptive, n (%)  probable neuropathic, n (%)  neuropathic, n (%) | 4 (20)  8 (40)  6 (30) |
| **SF-36** | |  |
| SF-36 physical composite | | 30 (24-37)/ 19-53 |
| SF-36 mental composite | | 50 (41-57)/ 31- 60 |

Abbreviations: IQR = Interquartile Range; CI = Confidence Interval; AQP4-IgG = aquaporin-4-immunoglobulin G; BDI-II = Beck´s Depression Inventory-II; EDSS = Expanded Disability Status Scale; NRS = numeric rating scale with values from 0 to 10 (0 no pain, 10 worst pain imaginable).

**eTable 3** Sample characteristics- mild depressive symptoms

|  | | **Median (IQR)/ (min. – max.)** |
| --- | --- | --- |
| **Demographic characteristics** | |  |
| Total number of patients | | 23 |
| Female Sex, n (%) | | 20 (87) |
| Age, in years | | 53 (48-61) |
| **Clinical characteristics** | |  |
| EDSS | | 4 (3.5-5)/ 0 – 8.5 |
| Disease duration, in years | | 7 (2-11) |
| Serostatus | | AQP4-IgG^+^ (n=19; 83%)  Double seronegative (n=4; 17%) |
| **CHANCE^NMO^ study parameters, Mean (95%CI)** | |  |
| Total annual cost of illness, € | | 52,588 (27,026-78,151) |
| Annual informal costs, € | | 19,147 (5,981-32,313) |
| Working patients, n (%) | | 5 (22) |
| Indirect costs, € | | 5,579 (1,703-9,456) |
| Early-retirement, n (%) | | 6 (26) |
| **PAIN & DEPRESSION study parameters** | |  |
| Patients with pain, n (%) | | 20 (87) |
| Median pain intensity according to NRS | | 5.0 (4.5-6) |
| Pain quality | nociceptive, n (%)  probable neuropathic, n (%)  neuropathic, n (%) | 6 (26)  6 (46)  8 (35) |
| **SF-36** | |  |
| SF-36 physical composite | | 31 (20-37)/ 14-49 |
| SF-36 mental composite | | 46 (38-55)/ 29- 62 |

Abbreviations: IQR = Interquartile Range; CI = Confidence Interval; AQP4-IgG = aquaporin-4-immunoglobulin G; BDI-II = Beck´s Depression Inventory-II; EDSS = Expanded Disability Status Scale; NRS = numeric rating scale with values from 0 to 10 (0 no pain, 10 worst pain imaginable).

**eTable 4** Sample characteristics- moderate depressive symptoms

|  | | **Median (IQR)/ (min. – max.)** |
| --- | --- | --- |
| **Demographic characteristics** | |  |
| Total number of patients | | 12 |
| Female Sex, n (%) | | 12 (100) |
| Age, in years | | 47 (41-59) |
| **Clinical characteristics** | |  |
| EDSS | | 4 (2.5-7.5)/ 1 – 7.5 |
| Disease duration, in years | | 6 (2,5-8) |
| Serostatus | | AQP4-IgG^+^ (n=12; 100%) |
| **CHANCE^NMO^ study parameters, Mean (95%CI)** | |  |
| Total annual cost of illness, € | | 68,738 (38,920-98,557) |
| Annual informal, costs € | | 36,724 (16,166-57,293) |
| Working patients, n (%) | | 5 (42) |
| Indirect costs, € | | 6,083 (0-13,291) |
| Early-retirement, n (%) | | 2 (17) |
| **PAIN & DEPRESSION study parameters** | |  |
| Patients with pain, n (%) | | 11 (92) |
| Median pain intensity according to NRS | | 5 (4-6) |
| Pain quality | nociceptive, n (%)  probable neuropathic, n (%)  neuropathic, n (%) | 2 (17)  1 (8.0)  8 (66) |
| **SF-36** | |  |
| SF-36 physical composite | | 32 (25-42)/ 19-54 |
| SF-36 mental composite | | 35 (28-43)/ 25- 52 |

Abbreviations: IQR = Interquartile Range; CI = Confidence Interval; AQP4-IgG = aquaporin-4-immunoglobulin G; BDI-II = Beck´s Depression Inventory-II; EDSS = Expanded Disability Status Scale; NRS = numeric rating scale with values from 0 to 10 (0 no pain, 10 worst pain imaginable).

**eTable 5** Sample characteristics- severe depressive symptoms

|  | | **Median (IQR)/ (min. – max.)** |
| --- | --- | --- |
| **Demographic characteristics** | |  |
| Total number of patients | | 9 |
| Female Sex, n (%) | | 8 (89) |
| Age, in years | | 50 (30-66) |
| **Clinical characteristics** | |  |
| EDSS | | 4 (3.5-6)/ 1.5 – 8.0 |
| Disease duration, in years | | 8,5 (2,5-14) |
| Serostatus | | AQP4-IgG^+^ (n=9; 100%) |
| **CHANCE^NMO^ study parameters, Mean (95%CI)** | |  |
| Total annual cost of illness, € | | 148,568 (65,123-232,014) |
| Annual informal costs, € | | 42,291 (15,191-69,391) |
| Working patients, n (%) | | 5 (56) |
| Indirect costs, € | | 63,317 (0-136,405) |
| Early-retirement, n (%) | | 1 (11) |
| **PAIN & DEPRESSION study parameters** | |  |
| Patients with pain, n (%) | | 8 (88) |
| Median pain intensity according to NRS | | 6.0 (4.5-6.5) |
| Pain quality | nociceptive, n (%)  probable neuropathic, n (%)  neuropathic, n (%) | 3 (33)  1 (11)  4 (44) |
| **SF-36** | |  |
| SF-36 physical composite | | 24 (20-28)/ 19-43 |
| SF-36 mental composite | | 30 (21.36)/ 25- 52 |

Abbreviations: IQR = Interquartile Range; CI = Confidence Interval; AQP4-IgG = aquaporin-4-immunoglobulin G; BDI-II = Beck´s Depression Inventory-II; EDSS = Expanded Disability Status Scale; NRS = numeric rating scale with values from 0 to 10 (0 no pain, 10 worst pain imaginable).
